# Effects of BNT162b2 mRNA Covid-19 vaccine on vascular function

**DOI:** 10.1371/journal.pone.0302512

**Published:** 2024-04-30

**Authors:** Takayuki Yamaji, Takahiro Harada, Yu Hashimoto, Yukiko Nakano, Masato Kajikawa, Kenichi Yoshimura, Chikara Goto, Yiming Han, Aya Mizobuchi, Farina Mohamad Yusoff, Shinji Kishimoto, Tatsuya Maruhashi, Ayumu Nakashima, Yukihito Higashi

**Affiliations:** 1 Department of Cardiovascular Medicine, Hiroshima University Graduate School of Biomedical Sciences, Hiroshima, Japan; 2 Division of Regeneration and Medicine, Medical Center for Translational and Clinical Research, Hiroshima University Hospital, Hiroshima, Japan; 3 Department of Biostatistics, Medical Center for Translational and Clinical Research, Hiroshima University Hospital, Hiroshima, Japan; 4 Department of Rehabilitation, Faculty of General Rehabilitation, Hiroshima International University, Hiroshima, Japan; 5 Department of Cardiovascular Regeneration and Medicine, Research Institute for Radiation Biology and Medicine, Hiroshima University, Hiroshima, Japan; 6 Department of Stem Cell Biology and Medicine, Hiroshima University Graduate School of Biomedical Sciences, Hiroshima, Japan; Sheikh Hasina National Institute of Burn & Plastic Surgery, BANGLADESH

## Abstract

The effects of Covid-19 vaccines on vascular function are still controversial. We evaluated the effects of BNT162b2 vaccine (BioNTech and Pfizer) on endothelial function assessed by flow-mediated vasodilation (FMD) and vascular smooth muscle function assessed by nitroglycerine-induced vasodilation (NID). This study was a prospective observational study. A total of 23 medical staff at Hiroshima University Hospital were enrolled in this study. FMD and NID were measured before vaccination and two weeks and six months after the 2^nd^ dose of vaccination. FMD was significantly smaller two weeks after the 2^nd^ dose of vaccination than before vaccination (6.5±2.4% and 8.2±2.6%, p = 0.03). FMD was significantly larger at six months than at two weeks after the 2^nd^ dose of vaccination (8.2±3.0% and 6.5±2.4%, p = 0.03). There was no significant difference between FMD before vaccination and that at six months after the 2^nd^ dose of vaccination (8.2±2.6% to 8.2±3.0%, p = 0.96). NID values were similar before vaccination and at two weeks, and six months after vaccination (p = 0.89). The BNT162b2 Covid-19 vaccine temporally impaired endothelial function but not vascular smooth muscle function, and the impaired endothelial function returned to the baseline level within six months after vaccination.

## Introduction

Coronavirus disease 2019 (COVID-19) is caused by severe acute respiratory syndrome coronavirus 2 (SARS-COV-2), which was found in China in 2019. The virus spread rapidly, and the World Health Organization declared a pandemic on March 11, 2020. As of April 4. 2022, about 490 million people worldwide have been infected and about six million people have died from COVID-19 [1]. Acute pneumonia is a major symptom of COVID-19, but SARS-COV-2 can affect various organs. SARS-COV-2 infection leads to various diseases such as taste dysfunction, meningitis, myocarditis, and arterial and venous thromboembolism [2–5]. The mechanisms by which COVID-19 leads to thrombosis and thromboembolism have remained unclear. It has been thought that endothelial dysfunction potentially contributes to thrombosis events [6].

Endothelial dysfunction caused by a decrease in nitric oxide (NO) bioavailability through imbalances between pro-inflammation and anti-inflammation and pro-oxidative stress and anti-oxidative stress is the first step of atherosclerosis [7–9]. Atherosclerosis progresses asymptomatically, finally leading to cardiovascular (CV) events [7, 10]. Rapid endothelial dysfunction also may contribute to thrombosis and thromboembolism [11]. Measurement of flow-mediated vasodilation (FMD) in the brachial artery is the most popular noninvasive tool for assessment of endothelial function. It has been shown that every 1% decrease in FMD is associated with a 10% increase in the risk of CV events [12]. On the other hand, nitroglycerine-induced vasodilation (NID) reflects vascular smooth muscle function.

The effectiveness of Covid vaccines have been established by clinical trials and real-world data [13, 14]. BNT162b2 (Comirnaty®; BioNTech and Pfizer) is one of the nucleoside-modified messenger ribonucleic acid (mRNA) vaccines for COVID-19. In Japan, 80% of subjects have received the 2^nd^ dose vaccine as of Mar. 31. 2022. In America, 66% of subjects have received the 2^nd^ dose vaccines as of Mar. 31. 2022 [15]. However, the number of new people receiving vaccination seems to have reached a plateau [15]. One reason for the lack of progress in vaccination may lie in concerns about serious adverse effects including CV events. Currently, there are no data on the long-term adverse effects of vaccines since BNT 162b2 vaccination started in December 2020. Therefore, in the present study, we assessed the effects of BNT162b2 vaccines on vascular function using FMD and NID as surrogate markers for CV events.

## Methods

### Study patients

A total of 23 medical staff (16 men and 7 women; aged 25 to 59 years) at Hiroshima University Hospital were enrolled in this study between February 2021 and January 2022. Hypertension was defined as the use of antihypertensive drugs or systolic blood pressure of more than 140 mm Hg or diastolic blood pressure of more than 90 mm Hg measured in a sitting position on at least 3 occasions. Dyslipidemia was defined according to the third report of the National Cholesterol Education Program [16]. Diabetes mellitus was defined according to the American Diabetes Association recommendation [17]. All methods were performed in accordance with the relevant guidelines and regulations. The Ethics Committee of Hiroshima University approved the study protocol. Written informed consent for participation in this study was obtained from all participants. The protocol was registered in the University Hospital Medical Information Network Clinical Trials Registry (UMIN000003409).

### Study protocol

This study was a single-center prospective interventional study at Hiroshima University. All of the subjects received two vaccinations (BNT162b2 mRNA vaccine; BioNTech and Pfizer). FMD and NID were measured before vaccination and two weeks and six months after the 2^nd^ dose of vaccination. The subjects fasted overnight and abstained from smoking, drinking alcohol, and taking antioxidant vitamins and caffeine for at least 12 hours before the study. Each of the participants was kept in a supine position in a quiet, dark, air-conditioned room (constant temperature of 22°C to 25°C) throughout the study. After maintaining the supine position for 30 minutes, FMD and NID were measured. The observers were blind to the form of examination.

### Measurements of FMD and NID

A high-resolution linear artery transducer was coupled to computer-assisted analysis software (UNEXEF18G, UNEX Co., Nagoya, Japan) that used an automated edge detection system to measure the brachial artery diameter [18]. The additional information is available in the supplemental method.

### Statistical analysis

Results are presented as means±SD. All reported probability values were 2-sided, and a probability value of <0.05 was considered statistically significant. Categorical values were compared by means of the chi-square test. FMD and NID were compared by using ANOVA for repeated measures with Fisher`s least significant difference by post hoc test. All data were processed using JMP Pro. Ver 14.0 software (SAS Institute, Cary, NC, USA)

## Results

### Baseline characteristics of the subjects

The baseline characteristics of the 23 subjects are summarized in Table 1. The mean age of the subjects was 36 years. The 23 subjects included 16 men. One subject had hypertension and 2 subjects had dyslipidemia.

**Table 1:** Clinical characteristics of subjects.

Variables	Total (n = 23)	No risk (n = 21)	At risk (n = 2)	P value
Age, yr	35±7	36±8	35±3	0.87
Men, n (%)	16 (59.3)	14 (66.7)	2 (100)	0.33
Body mass index, kg/m^2^	21.3±3.2	21.1±2.9	27.0±5.1	0.01
Heart rate, bpm	71±8	72±9	70±1	0.80
Systolic blood pressure, mmHg	118±15	116±12	147±31	0.005
Diastolic blood pressure, mmHg	74±10	74±9	90±23	0.04
Medical history, n (%)				
Hypertension	1 (4.3)	0 (0)	1 (50)	<0.001
Dyslipidemia	2 (8.7)	0 (0)	2(100)	<0.001
Diabetes mellitus	0	0 (0)	0 (0)	N.S.
Current smoker, n (%)	0	0 (0)	0 (0)	N.S.
Medication, n (%)				
Antihypertensive drugs	1 (4.3)	0 (0)	1 (50)	<0.001
Lipid lowering drugs	2 (8.7)	0 (0)	2(100)	<0.001
Anti-diabetic drugs	0	0 (0)	0 (0)	N.S.

### Effect of BNT162b2 vaccine on endothelial function

**Fig 1:**
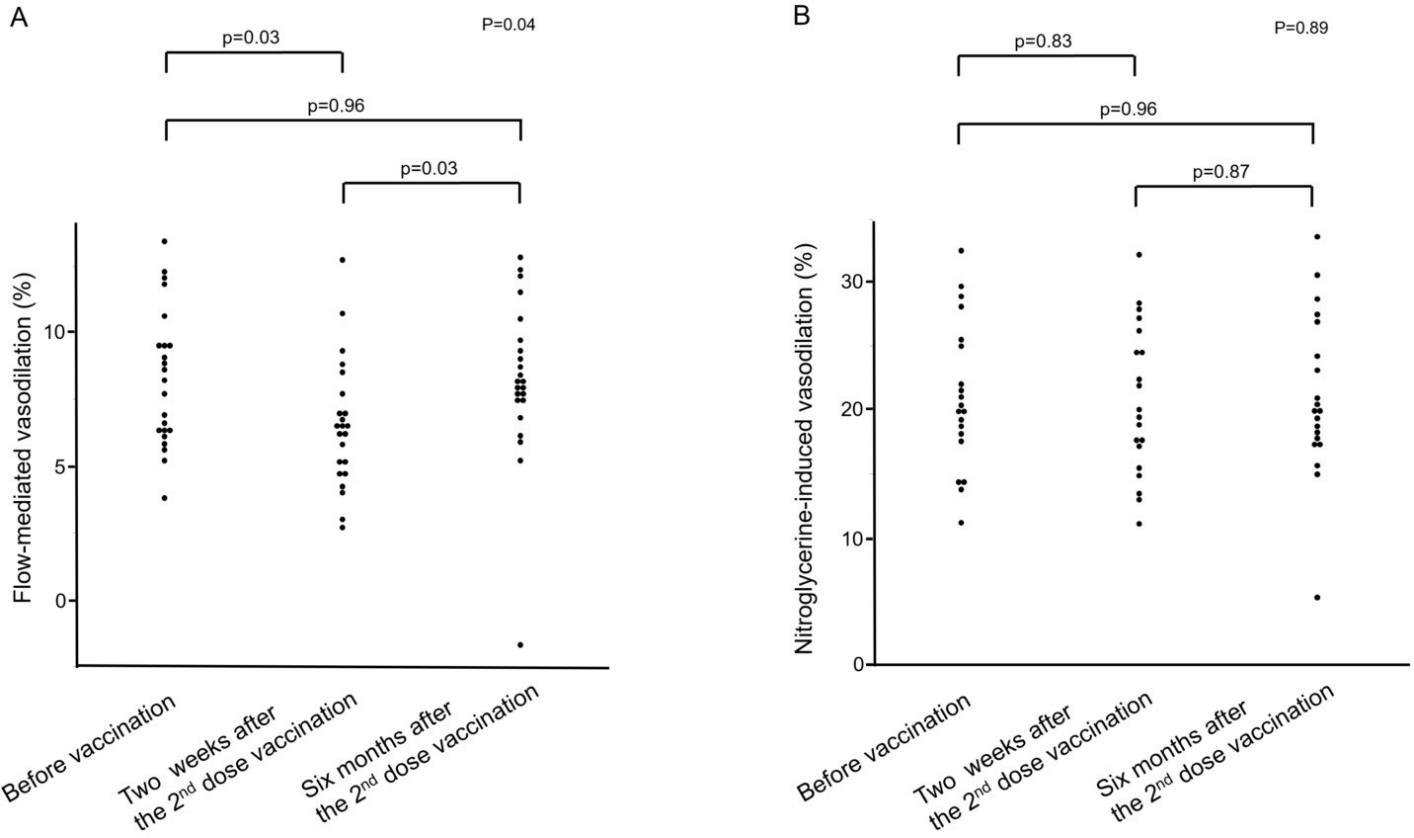
Dot graphs show effects of the BNT162b2 mRNA Covid-19 vaccine on flow-mediated vasodilation (A) and nitroglycerine-induced vasodilation (B) before vaccination and at two weeks and six months after the 2^nd^ dose of vaccination.

Fig 1A shows changes in FMD before vaccination and during the follow-up periods after vaccination. FMD was significantly smaller at two weeks after the 2^nd^ dose of vaccination than before vaccination (6.5±2.4% and 8.2±2.6%, p = 0.03), and FMD was significantly larger at six months than at two weeks after the 2^nd^ dose of vaccination (8.2±3.0% and 6.5±2.4%, p = 0.03). There was no significant difference between FMD before vaccination and FMD at six months after the 2^nd^ dose of vaccination (8.2±2.6% to 8.2±3.0%, p = 0.96).

### Effect of BNT162b2 vaccine on vascular smooth muscle function

Fig 1B shows changes in NID before vaccination and during the follow-up periods after vaccination. NID values were similar before vaccination and at two weeks and six months after the 2^nd^ dose of vaccination (21.1±5.8%, 20.6±5.9%, and 21.0±6.4%, respectively, p = 0.89).

## Discussion

We demonstrated for the first time that endothelial function was impaired by BNT162b2 mRNA Covid-19 vaccination and returned to the baseline level within six months after vaccination and that BNT162b2 mRNA Covid-19 vaccination did not alter vascular smooth muscle function.

The efficacy of the BNT162b2 mRNA Covid-19 vaccine is well established. A previous study showed that two doses of BNT162b2 vaccine reduced the occurrence of Covid-19 by 95% compared with that in a placebo group [14]. However, various adverse events have been reported to occur within a short period of time after BNT162b2 mRNA Covid-19 vaccination [14]. A few subjects had severe adverse events after receiving the BNT162b2 mRNA Covid-19 vaccine [14]. Mevorach et al. reported that the incidence of myocarditis increased after the 2^nd^ dose of vaccination in young male subjects [19]. However, the long-term effects of the BNT162b2 mRNA Covid-19 vaccine on organ damage and incidence of CV events are still uncertain. Several studies including our study have shown that endothelial function assessed by FMD is a surrogate marker for organ damage and cardiovascular events [20, 21]. FMD was significantly smaller at two weeks after the 2^nd^ dose of vaccination than before vaccination. FMD had returned to the baseline level at six months after the 2^nd^ dose of vaccination. NID before vaccination was similar to that during the follow-up periods after vaccination. These results suggest that the BNT162b2 mRNA Covid-19 vaccine impairs endothelial function but not vascular smooth muscle function early post-vaccination and that the BNT162b2 mRNA Covid-19 vaccine-induced endothelial dysfunction is restored within six months after vaccination.

In the present study, we confirmed that the BNT162b2 mRNA Covid-19 vaccine impaired endothelial function in relatively healthy subjects and that the BNT162b2 mRNA Covid-19 vaccine-induced endothelial dysfunction was restored to the baseline level within six months after the 2^nd^ dose of vaccination. Although long-term effects of the BNT162b2 mRNA Covid-19 vaccine on CV events may be limited, endothelial dysfunction may be, at least in part, involved in thrombotic events that develop relatively early after Covid-19 vaccination. In addition, we cannot deny the possibility that harmful effects of the BNT162b2 mRNA Covid-19 vaccine on vascular function are stronger in patients with advanced atherosclerosis than in healthy individuals and that Covid-19 vaccination-induced impairment of vascular function is prolonged in those patients. Future studies are needed to confirm the short-term and long-term effects of Covid-19 vaccination on vascular function in individuals with high cardiovascular risk factors and patients with cardiovascular disease.

This study has potential limitations. First, this study was a single-center study with a small sample size and was conducted in only medical staff. In addition, it is clinically important to evaluate the effects of vaccination on vascular function in patients with cardiovascular risk factors including hypertension, dyslipidemia, and diabetes mellitus. Unfortunately, only one subject with dyslipidemia and one subject with dyslipidemia and hypertension were included in the present study. Future studies are needed to confirm the roles of vaccination in vascular function in patients who have cardiovascular risk factors and cardiovascular disease in a large study population. However, we confirmed significant changes in FMD during a six-month follow-up period after vaccination. Second, we did not have information on blood biochemistry at each time point when vascular function was assessed. Measurements of inflammatory markers, antibodies to Covid-19 vaccine and immune markers would enable more specific conclusions concerning the roles of Covid-19 vaccine in vascular function to be drawn. Third, in the present study, we could not use placebo due to ethical issues.

## Conclusions

In conclusion, BNT162b2 mRNA Covid-19 vaccine temporally impaired endothelial function and the impaired endothelial function returned to the baseline level within six months after the 2^nd^ dose of vaccination. Although long-term effects of BNT162b2 mRNA Covid-19 vaccine on CV events may be limited, endothelial dysfunction may be, at least in part, involved in thrombotic events that develop relatively early after Covid-19 vaccination.

## Supporting information

S1 File (DOCX)
