# Supplementary material for: Effects of BNT162b2 mRNA Covid-19 vaccine on vascular function
Source: PLoS One. 2024 Apr 30;19(4):e0302512. doi: 10.1371/journal.pone.0302512 (PMC11060586; doi:10.1371/journal.pone.0302512)
Supplement: S1 File — (DOCX) [file pone.0302512.s001.docx]

**Supplemental Data**

**Effects of BNT162b2 mRNA Covid-19 Vaccine on Vascular Function**

Running title: Covid-19 vaccine and vascular function

Takayuki Yamaji, MD, PhD;^1^ Takahiro Harada, MD, PhD;^1^ Yu Hashimoto, MD;^1^ Yukiko Nakano, MD, PhD;^1^ Masato Kajikawa, MD, PhD;^2^ Kenichi Yoshimura, PhD;^2,3^ Chikara Goto, PhD; ^4^ Yiming Han, MS;^5^ Aya Mizobuchi, MS;^5^ Farina Mohamad Yusoff, MD, PhD;^5^ Shinji Kishimoto, MD, PhD;^5^ Tatsuya Maruhashi, MD, PhD;^5^ Ayumu Nakashima, MD, PhD;^6^ Yukihito Higashi, MD, PhD, FAHA^2,5^

^1^Department of Cardiovascular Medicine, Hiroshima University Graduate School of Biomedical Sciences, Hiroshima, Japan

^2^Division of Regeneration and Medicine, Medical Center for Translational and Clinical Research, Hiroshima University Hospital, Hiroshima, Japan

^3^Department of Biostatistics, Medical Center for Translational and Clinical Research, Hiroshima University Hospital, Hiroshima, Japan

^4^Department of Rehabilitation, Faculty of General Rehabilitation, Hiroshima International University, Hiroshima, Japan

^5^Department of Cardiovascular Regeneration and Medicine, Research Institute for Radiation Biology and Medicine, Hiroshima University, Hiroshima, Japan

^6^Department of Stem Cell Biology and Medicine, Hiroshima University Graduate School of Biomedical Sciences, Hiroshima, Japan

**Financial support**

Grant-in-Aid for Scientific Research from the Ministry of Education, Science and Culture of Japan　(18590815 and 21590898 to Y.Higashi).

**Conflict of interest**

All authors have no conflicts of interests to report.

**Address for correspondence:** Yukihito Higashi, MD, PhD, FAHA

Department of Cardiovascular Regeneration and Medicine,

Research Institute for Radiation Biology and Medicine, Hiroshima University

1-2-3 Kasumi, Minami-ku, Hiroshima 734-8551, Japan

Phone: +81-82-257-5831 Fax: +81-82-257-5831

E-mail: [yhigashi@hiroshima-u.ac.jp](mailto:yhigashi@hiroshima-u.ac.jp)

**Supplemental Methods**

**Measurement of FMD and NID**

A blood pressure cuff was placed around the forearm of each subject. The brachial artery was scanned longitudinally 5 to 10 cm above the elbow. When the clearest B-mode image of the anterior and posterior intimal interfaces between the lumen and vessel wall was obtained, the transducer was held at the same point throughout the scan by using a special probe holder (UNEX Co.) to ensure consistent imaging. Depth and gain settings were set to optimize the images of the arterial lumen wall interface. When the tracking gate was placed on the intima, the artery diameter was automatically tracked, and the waveform of the diameter changes over the cardiac cycle was displayed in real time using the FMD mode of the tracking system. This allowed the ultrasound images to be optimized at the start of the scan and the transducer position to be adjusted immediately for optimal tracking performance throughout the scan. Pulsed Doppler flow was assessed at baseline and during peak hyperemic flow, which was confirmed to occur within 15 seconds after cuff deflation. Blood flow velocity was calculated from the color Doppler data and was displayed as a waveform in real time. Baseline longitudinal images of the artery were acquired for 30 seconds, and then the blood pressure cuff was inflated to 50 mm Hg above systolic pressure for five minutes. The longitudinal image of the artery was recorded continuously until five minutes after cuff deflation. Pulsed Doppler velocity signals were obtained for 20 sec at baseline and for 10 sec immediately after cuff deflation. Changes in brachial artery diameter were immediately expressed as percentage changes relative to the vessel diameter before cuff inflation. FMD was automatically calculated as the percentage change in peak vessel diameter from the baseline value. The percentage FMD [(peak diameter - baseline diameter)/baseline diameter] was used for analysis. Blood flow volume was calculated by multiplying the Doppler flow velocity (corrected for the angle) by heart rate and vessel cross-sectional area (-r^2^). Reactive hyperemia was calculated as the maximum percentage increase in the flow after cuff deflation compared with the baseline flow.

The response to nitroglycerine was used for the assessment of endothelium-independent vasodilation.[1] After acquiring baseline rest images for 30 seconds, a sublingual tablet (nitroglycerine, 75 µg) was given, and imaging of the artery was done continuously for five minutes. NID was automatically calculated as the percentage change in peak vessel diameter from the baseline. The percentage NID [(peak diameter - baseline diameter)/baseline diameter] was used for analysis. The inter- and intra-assay coefficients of variation for the brachial artery diameter in our laboratory were 1.6% and 1.4%, respectively.

1. Maruhashi T, Soga J, Fujimura N, Idei N, Mikami S, Iwamoto Y, et al. Nitroglycerine-induced vasodilation for assessment of vascular function: a comparison with flow-mediated vasodilation. Arterioscler Thromb Vasc Biol. 2013;33(6):1401-8. Epub 2013/03/23. doi: 10.1161/atvbaha.112.300934. PubMed PMID: 23520168.
